# Effects of the COVID-19 pandemic on the pediatric emergency department: a single institution experience

**DOI:** 10.1186/s40621-022-00401-w

**Published:** 2022-12-21

**Authors:** Isabella V. Masler, Nipam Shah, Shea A. Duerring, Kathy R. Monroe

**Affiliations:** 1 Department of Pediatrics, University of Alabama at Birmingham, Birmingham, AL 35223 USA; 2 Division of Pediatric Emergency Medicine, Department of Pediatrics, University of Alabama at Birmingham, Birmingham, AL 35223 USA

**Keywords:** COVID-19, Pediatric, Injury prevention, Emergency department, Pandemic, Trends

## Abstract

**Background:**

The COVID-19 pandemic resulted in drastic decreases in volume for most pediatric emergency departments (ED). Injuries have persisted and there is concern that injuries may have increased during the pandemic. This study evaluates the impact of the COVID-19 pandemic on ED patient trends at a freestanding children’s hospital.

**Results:**

Despite an average annual increase of 1100 patients per year between 2017 and 2019, this ED saw a decrease of over 25,000 patients in 2020. The number of trauma alerts increased from 341 in 2017 to 571 in 2020 and those numbers remained stable (568–571) in 2020 compared to 2019. The percent of total volume accounted for by trauma alerts increased from 0.65 to 1.2% between 2019 and 2020 (following the trend of 0.48% in 2017 to 0.56% in 2018). Historically, motor vehicle crashes account for the majority of the trauma alerts, though the number of trauma alerts from firearm-related injuries increased from 36 per year in 2018 to 44 in 2019 to 66 (12% of total trauma alerts) in 2020.

**Conclusions:**

While total volumes of patients being seen decreased, the number of trauma alerts remained stable resulting in an increased percentage of trauma alert patients. This indicates that severe injuries requiring trauma alert activation did not diminish during the pandemic. These trends have implications for prevention as well as implications for ED staffing. Changing trends in types of severe injuries are noted.

## Background

Severe Acute Respiratory Syndrome Coronavirus 2 (SARS-CoV-2) emerged in China in late 2019 and was declared a pandemic on March 11, 2020, by the World Health Organization (World Health Organization 2020). Adult healthcare systems faced tremendous challenges in patient volume and acuity; however, morbidity and mortality among children was low and pediatric emergency departments seemed to have been spared the same overwhelming influx of patients (Flores et al. 2020; American Academy of Pediatrics 2020; CDC 2019; Sisk et al. 2020). Efforts to curb disease transmission such as shelter-in-place orders and social distancing have negatively affected delivery of primary healthcare in the USA (Chang et al. 2021; Palinkas et al. 2021). Additionally, in-person school closures and cancellation of typical activities for children have had further impacts on disease patterns in children and presentations for emergency care (Bartsch et al. 2020; Pines et al. 2020; Chaiyachati et al. 2020; Even et al. 2020; Kover et al. 2020). The impact of these factors and the COVID-19 epidemic on pediatric emergency departments has yet to be fully understood. Several studies have shown that despite decreased pediatric emergency department volume, acuity and trauma have increased along with specific increases in several specific diseases including injuries, non-accidental trauma, mental health visits, and ingestions (Pines et al. 2020; Chaiyachati et al. 2020; Even et al. 2020; Kover et al. 2020; DeLaroche et al. 2021; Sokoloff et al. 2021). The purpose of this study is to evaluate the COVID-19 epidemic’s varied impact in emergency department visits and admission trends on our tertiary care pediatric emergency department and level 1 trauma center.

## Results

**Fig. 1:**
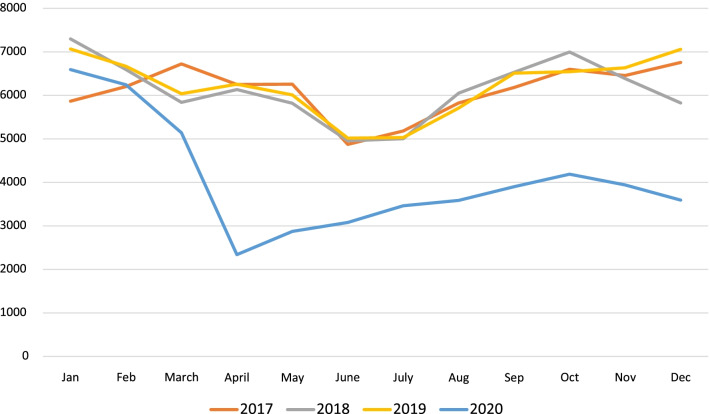
Monthly trends of overall number of Emergency Department visits for the years 2017–2020. Orange line represents 2017, gray line represents 2018, yellow line represents 2019, and blue line represents 2020

**Fig. 2:**
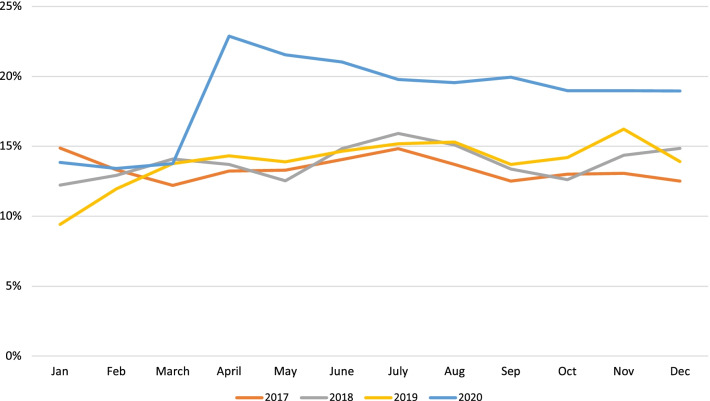
Monthly trends of the percentage of patients admitted from the Emergency Department for the years 2017–2020. Orange line represents 2017, gray line represents 2018, yellow line represents 2019, and blue line represents 2020

**Fig. 3:**
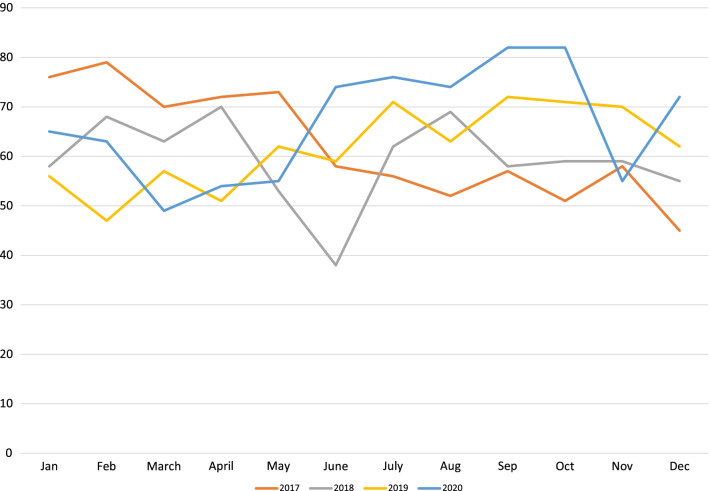
Monthly trends of overall admission numbers to the inpatient psychiatry service from the ED for the years 2017–2020. Orange line represents 2017, gray line represents 2018, yellow line represents 2019, and blue line represents 2020

Overall ED volume for this institution decreased by 34% (95% CI of − 45.7 to − 23.5%) from a high of 74,513 (2019) to 48,924 (2020). The overall volume for 2020 decreased by 25,589, in contrast to trend of volume from 2017 through 2019, which had previously shown a steady increase in overall volume by an additional 1100 patients per year (Fig. 1). Average yearly admission rates to the hospital from the ED increased from 13.4 to 13.9% in 2017–2019 to 18.6% in 2020 (Table 1). Peak admittance occurred in April 2020 with an admission rate as high as 22.9% (Fig. 2). When comparing the types of admissions, the institution saw a linear increase in combined admission rates to the Pediatric Intensive Care Unit and Intermediate Care Unit from a high of 23.7% in 2019 to 25.4% in 2020 with a corresponding 6% decrease (95% CI of − 13.8 to 3.4%) in admission rates to Acute Care Floors (Table 1). Additionally, the rate of admission to the inpatient psychiatry unit increased, with an average yearly admission rate of 7.2–7.7% in 2017–2019 compared to 9.3% in 2020, with a stark 29% (95% CI of 13.8–46.9%) increase comparing 2019 to 2020 (Table 1, Fig. 3). The number of patients transported via EMS to our institution went from 3248 in 2019 to 4422 in 2020, with 2034 of those patients in 2020 being transferred from outside facilities.

**Table 1:** Pediatric emergency department volume numbers and admission rates by year with percent changes and respective 95% confidence intervals

	2017	2018	2019	2020	% change (95% CI of % change) 2019–2020	% change (95% CI of % change) 2018–2020	% change (95% CI of % change) 2017–2020
Overall ED volume	73,144	73,404	74,513	48,924	**− 34.3%** **(− 45.7%, − 23.5%)**	**− 33.3%** **(− 44.6%, − 23.1%)**	**− 33.1%** **(− 46.3%, − 19.5%)**
Admission rates	13.3% (9745)	13.8% (10,119)	13.8% (10,272)	17.7% (8645)	**+ 28.3%** **(22.7%, 45.3%)**	**+ 28.3%** **(19.3%, 48.7%)**	**+ 33.1%** **(23.4%, 54.8%)**
Critical care admission rates*	21.2% (2063)	21.9% (2216)	23.7% (2434)	25.4% (2194)	+ 7.2% (− 1.4%, 17.8%)	**+ 15.9%** **(10.7%, 22.1%)**	**+ 19.8** **(14.1%, 27.1%)**
ACB admission rates*	61.6% (6007)	61.9% (6267)	60.5% (6210)	56.8% (4906)	− 6.1% (− 13.8%, 3.4%)	**− 8.2%** **(− 12.6%, − 4.6%)**	**− 7.8%** **(− 13.0, − 3.3%%)**
Percent of ED psychiatric visits	3.4% (2512)	4.1% (3006)	4.9% (3679)	5.9% (2932)	**+ 20.4%** **(7.8%, 50.6%)**	**+ 43.9%** **(32.9%, 72.9%)**	**+ 73.5%** **(55.1%, 105.4%)**
Psychiatry admission rates	7.7% (747)	7.0% (712)	7.2% (741)	9.3% (801)	**+ 29.2 (13.8%, 46.9%)**	**+ 32.8%** **(13.2%, 59.3%)**	**+ 20.7%** **(2.4%, 52.6%)**

If 95% confidence interval does not cross zero, the difference is statistically significant. Values marked in bold are significant

*Denominator is total number of admissions

**Fig. 4:**
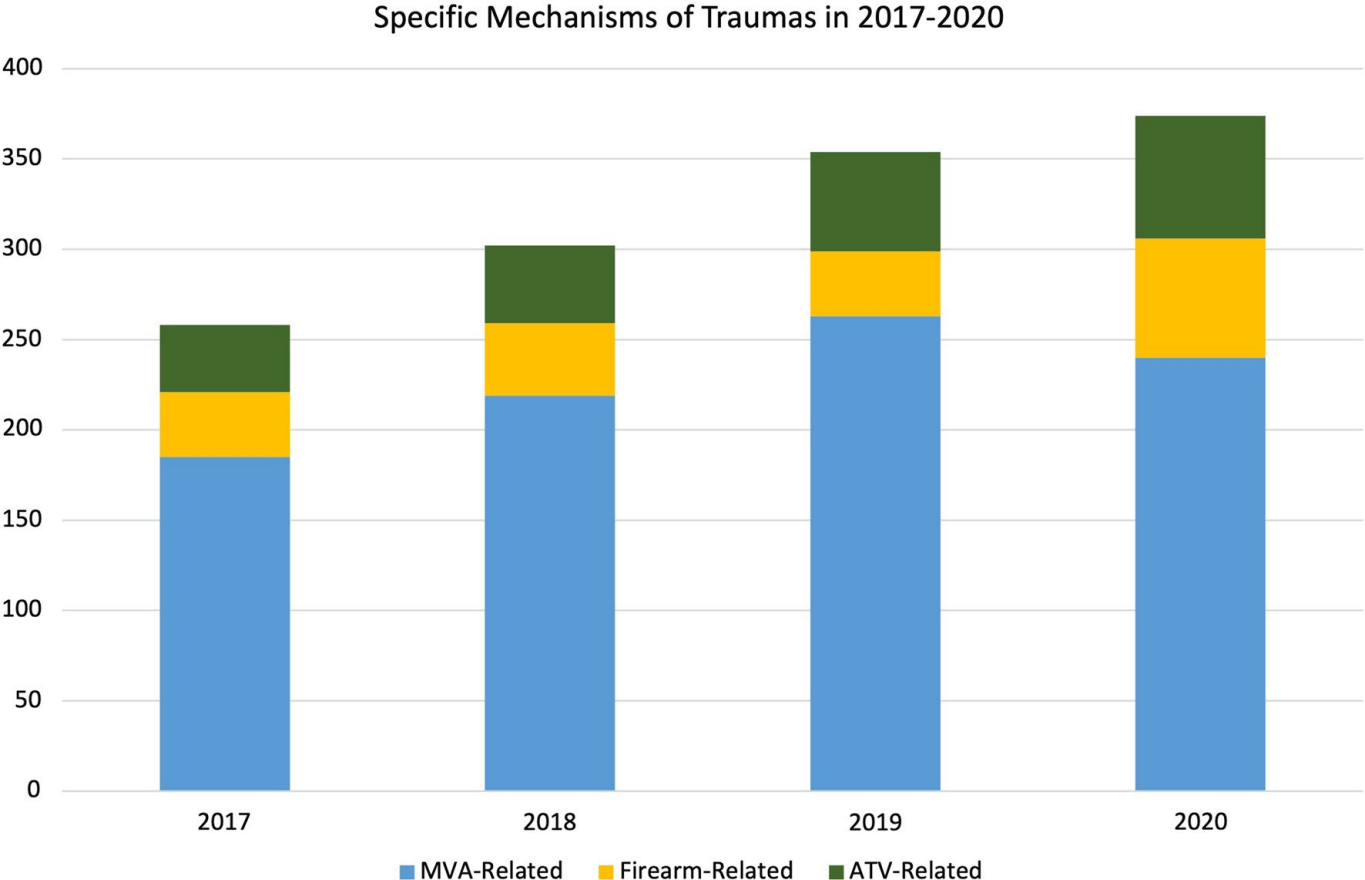
Yearly trends of specific mechanisms of injuries that met trauma activation criteria in 2017–2020. Specific mechanisms included MVC-, firearm-, and ATV-related incidents. Blue bar represents MVC-related, yellow bar represents firearm-related, and green bar represents ATV-related

The total number of injury-related ED visits decreased from 12, 654 (2019) to 9977 (2020) but remained increased compared to 6164 (2017) and 5590 (2018). The percent of total ED patient volume accounted for by trauma alerts increased from 0.65 to 1.2% in the 2019–2020 year, in line with the trend of increasing trauma activation rates from 0.48% in 2017 to 0.56% in 2018. Injuries related to motor vehicle accidents continued to account for the majority of the trauma alerts seen at this institution. However, there was an increase in firearm-related injuries from 36 in 2018 to 44 in 2019 to 66 in 2020 (Fig. 4). These types of injuries accounted for 12% of total trauma alerts in 2020, which was a steady increase from the 7–10% in 2017–2019.

## Discussion

The SARS-CoV-2 pandemic led to a reduction in ED visits across the country in both the adult and pediatric populations. In line with recent studies, this institution saw significantly decreased patient volumes in 2020 compared to each previous year (Palinkas et al. 2021; Bartsch et al. 2020; Pines et al. 2020). Overall patient volumes sharply decreased starting in February 2020 and reached the lowest numbers in March with volumes remaining below the pre-pandemic levels throughout the year. Stay-at-home quarantine and social distancing orders likely contributed to the decreases in patient volumes seen in the early stages of the pandemic, while a surge in primary care telehealth medicine visits may have kept volumes low as the state began to ease its social distancing guidelines in the later months of 2020. Our study adds to the growing body of research that illustrates the opportunities that ED providers have for education on injury prevention because injury numbers remained stable despite a global pandemic, as well as the implications for appropriate ED staffing.

Despite the decrease in overall patient volumes, the acuity of patients seen in the ED remained high, as shown by the markedly increased rate of admission to the hospital. The peak admission rates occurred in the months immediately following the start of the pandemic (March 2020) and the yearly admission rate for 2020 was elevated compared to previous years. Particularly, the admission rates to the intensive care and step-down care units were elevated in 2020 compared to 2019 with a corresponding reduction in the acute care floor admission rates. Recent studies also demonstrate the decrease in lower acuity visits that typically compose a majority of the volume of patient seen in the pediatric emergency department, there is still little data that exists currently that demonstrates the substantial increase in high acuity patients admitted to the hospital that required intensive level care (Bartsch et al. 2020; Pines et al. 2020; Even et al. 2020). The decrease in low acuity visits possibly represents hesitancy from caregivers to seek medical attention once stay-at-home orders were issued in March of 2020. Further correlations can be seen between the reduction in face-to-face interactions and close contacts of school-aged children and the reduction in the incidence of communicable diseases especially with respiratory viruses. Additionally, other studies note an increase in telemedicine visits, specifically for routine check-ups and typical sick visits, which could have contributed to the decreases seen in lower acuity visits.

Looking specifically at injury-related ED visits, there was an overall decrease in injury numbers; however, the number of trauma alert activations rose, indicating that the types of injuries that were seen during 2020 required more intensive care and medical resources. The Covid-19 pandemic seemed to have varied effects on specific injury types and trauma activations that presented to the ED. Notably, there was an increase in the number of MVC- and firearm-related trauma activations consistent with other recent literature (Bartsch et al. 2020; Chaiyachati et al. 2020). These trends represent an important area of public health that can be addressed to emphasize education and policies to reduce modifiable risk factors that contribute to these types of traumas. During the pandemic, ED physicians have been in a unique position to play a larger role in providing anticipatory guidance due to decreased availability of health care during times of limited contact with primary healthcare providers who usually address risk factors that help prevent injuries and traumas such as firearm and MVC-related events.

It is worth noting that there was an increase in the percentage of ED visits for psychiatric reasons and the number of inpatient psychiatry admissions in 2020 compared to previous years. While this trend has been seen in the adult populations, there have been few studies that illustrate the same phenomenon in the pediatric population (Bartsch et al. 2020). Notably, our study demonstrates a predictable seasonality to psychiatry admissions, with peak admission numbers during the winter months and the lowest admission numbers occurring during the summer months. During 2020, our peak admission numbers were shifted to the months immediately following the institution of the statewide stay-at-home orders and remained overall elevated through the remainder of the calendar year. This correlation suggests that the social distancing and disruption of the normal school year may have negatively impacted children and adolescent’s mental health.

### Limitations

Data were obtained from one institution over a 4 year period. This may not be generalizable to other institutions; however, it seems to be consistent with other reported findings. Additionally, initial data analysis began in the middle of 2021, so this study is limited to the pre-pandemic and early pandemic effects on the institution. Future directions for this study will include updated ED numbers from 2021 to assess how the surges of Covid variants affected the trends noted here. Because this analysis is limited to pediatric ED data, patients may have sought care elsewhere such as urgent cares or even adult EDs. This is less likely because we are the only Level 1 pediatric trauma center in the state, though our trauma data may actually underestimate the statewide trends because pediatric patients are evaluated in other institutions. Likewise it is unlikely to be a significant factor for the critically ill patients as our institution serves as the tertiary referral center for critically ill patients for the state.

### Conclusions

The results from this study suggest that the overall use of our pediatric emergency department was significantly reduced; however, this trend was largely due to the reduction in the high volumes of lower acuity ED visits. The acuity of the ED visits increased as evidenced by the increase in critical care admissions, EMS transfers, and trauma activation rates in 2020 compared to previous years. Additionally, the increase in psychiatric-related ED visits and hospital admissions highlights the ongoing need for available resources that deliver primary mental health care especially as the delivery of outpatient health care is shifting toward more telemedicine. These trends argue for the importance of maintaining adequate medical resources and appropriate staffing in pediatric EDs to meet the needs of the increased percentage of higher acuity visits, as well as the psychiatric-related visits that require considerable resources specifically ED beds for boarding and additional medical staff for safety. This study demonstrates that preventable injuries are a constant public health concern in the pediatric population and highlight the need to continue focusing our efforts on injury prevention, despite a worldwide pandemic.

## Methods

This retrospective time series study was conducted at the University of Alabama Birmingham with data coming from three sources: (1) State Office of EMS (emergency medical service records), (2) Hospital emergency department (ED) visits and admission data, and 3. Hospital trauma database. The hospital is a free standing children’s hospital with an annual emergency department census around 75,000 prior to the 2020 pandemic year. The hospital is the only state certified pediatric level I trauma center. Strict criteria exist for triggering trauma team activation into either level 1 or level 2 traumas. In alignment with previous studies that observed how the COVID-19 pandemic affected general trends in healthcare settings, 4 years of data from 2017 to 2020 were analyzed (Bartsch et al. 2020; Pines et al. 2020; Even et al. 2020; Kover et al. 2020). Specific data analyzed included total number of ED patients seen, number and percent of patients admitted to a critical care unit, number and percent of ED patients who met trauma activation criteria, and number of EMS transfers. Injury visits that did not meet the trauma alert criteria were excluded from the trauma numbers. One thing to note is that this hospital was never placed in “divert” status during the 2020 year. This study was deemed exempt by the institutional review board at the University of Alabama Birmingham since all data were de-identified and aggregate. Data were managed and analyzed using Microsoft Excel spreadsheet to create data tables that allowed direct comparisons of total numbers and created percentages for each category stated above. Comparisons for this descriptive analysis were done utilizing relative percent changes with confidence intervals of 95%.

## Data Availability

Data available on request.

## Abbreviations

ED: Emergency department
SARS-CoV-2: Severe Acute Respiratory Syndrome Coronavirus 2
